# The Eighth Central European Conference “Chemistry towards Biology”: Snapshot †

**DOI:** 10.3390/molecules21101381

**Published:** 2016-10-17

**Authors:** András Perczel, Atanas G. Atanasov, Vladimír Sklenář, Jiří Nováček, Veronika Papoušková, Pavel Kadeřávek, Lukáš Žídek, Henryk Kozłowski, Joanna Wątły, Aleksandra Hecel, Paulina Kołkowska, Jaroslav Koča, Radka Svobodová-Vařeková, Lukáš Pravda, David Sehnal, Vladimír Horský, Stanislav Geidl, Ricardo D. Enriz, Pavel Matějka, Adéla Jeništová, Marcela Dendisová, Alžběta Kokaislová, Volkmar Weissig, Mark Olsen, Aidan Coffey, Jude Ajuebor, Ruth Keary, Marta Sanz-Gaitero, Mark J. van Raaij, Olivia McAuliffe, Birgit Waltenberger, Andrei Mocan, Karel Šmejkal, Elke H. Heiss, Marc Diederich, Robert Musioł, Janez Košmrlj, Jarosław Polański, Josef Jampílek

**Affiliations:** 1 Laboratory of Structural Chemistry and Biology and MTA-ELTE Protein Modeling Research Group at the Institute of Chemistry, Eötvös Loránd University, 1518, 112, PO Box 32, H-1053 Budapest, Hungary; 2 Department of Pharmacognosy, Faculty of Life Sciences, University of Vienna, Althanstrasse 14, 1090 Vienna, Austria; elke.heiss@univie.ac.at; 3 Institute of Genetics and Animal Breeding of the Polish Academy of Sciences, ul. Postepu 36A, 05552 Jastrzebiec, Poland; 4 Central European Institute of Technology, Masaryk University, Kamenice 5, 62500 Brno, Czech Republic; vladimir.sklenar@ceitec.muni.cz (V.S.); jiri.novacek@ceitec.muni.cz (J.N.); veronika.papouskova@ceitec.muni.cz (V.P.); kada@ncbr.muni.cz (P.K.); lukas.zidek@ceitec.muni.cz (L.Z.); jkoca@ceitec.cz (J.K.); svobodova@chemi.muni.cz (R.S.-V.); luky.pravda@gmail.com (L.P.); david.sehnal@gmail.com (D.S.); vl.horsky@gmail.com (V.H.); geidl.stanislav@gmail.com (S.G.); 5 Department of Biological Chemistry, Faculty of Chemistry, University of Wroclaw, F. Joliot-Curie 14, 50-383 Wroclaw, Poland; henryk.kozlowski@chem.uni.wroc.pl (H.K.); joanna.watly@chem.uni.wroc.pl (J.W.); aleksandra.hecel@chem.uni.wroc.pl (A.H.); paulina.kolkowska@chem.uni.wroc.pl (P.K.); 6 National Centre for Biomolecular Research, Faculty of Science, Masaryk University, Kamenice 5, 62500 Brno, Czech Republic; 7 Facultad de Química, Bioquímica y Farmacia, Universidad Nacional de San Luis, Chacabuco 917, 5700 San Luis, Argentina; denriz@unsl.edu.ar; 8 Instituto Multidisciplinario de Investigaciones Biológicas de San Luis, Ejercito de Los Andes 950, 5700 San Luis, Argentina; 9 Department of Physical Chemistry, Faculty of Chemical Engineering, University of Chemistry and Technology, Technická 5, 16628 Prague 6, Czech republic; pavel.matejka@vscht.cz (P.M.); adela.jenistova@vscht.cz (A.J.); marcela.dendisova@vscht.cz (M.D.); alzbeta.kokaislova@vscht.cz (A.K.); 10 Department of Medical Chemistry, College of Pharmacy Glendale, Midwestern University, 19555 N. 59th Avenue, Glendale, 85308 AZ, USA; vweiss@midwestern.edu (V.W.); molsen@midwestern.edu (M.O.); 11 Department of Biological Sciences, Cork Institute of Technology, Bishopstown, T12 P928 Cork, Ireland; aidan.coffey@cit.ie (A.C.); jude.ajuebor@mycit.ie (J.A.); ruth.keary@mycit.ie (R.K.); 12 Departamento de Estructura de Macromoleculas, Centro Nacional de Biotecnologia, Calle Darwin 3, 28049 Madrid, Spain; msanz@cnb.csic.es (M.S.-G.) mjvanraaij@cnb.csic.es (M.J.v.R.); 13 Biotechnology Department, Teagasc, Moorepark Food Research Centre, Fermoy, P61 C996 Co. Cork, Ireland; olivia.mcauliffe@teagasc.ie; 14 Department of Pharmacognosy, Institute of Pharmacy, University of Innsbruck, Innrain 80-82/IV, 6020 Innsbruck, Austria; birgit.waltenberger@uibk.ac.at; 15 Department of Pharmaceutical Botany, Iuliu Hațieganu University of Medicine and Pharmacy, 8 Victor Babes, 400012 Cluj-Napoca, Romania; mocan.andrei@umfcluj.ro; 16 Department of Natural Drugs, Faculty of Pharmacy, University of Veterinary and Pharmaceutical Sciences Brno, Palackého 1, 61242 Brno, Czech Republic; karel.mejkal@post.cz; 17 Department of Pharmacy, College of Pharmacy, Seoul National University, 1 Gwanak-ro, 08826 Seoul, Korea; marcdiederich@snu.ac.kr; 18 Institute of Chemistry, University of Silesia, Szkolna 9, 40007 Katowice, Poland; robert.musiol@us.edu.pl (R.M.); polanski@us.edu.pl (J.P.); 19 Faculty of Chemistry and Chemical Technology, University of Ljubljana, Večna pot 113, 1000 Ljubljana, Slovenia; janez.kosmrlj@fkkt.uni-lj.si; 20 Department of Pharmaceutical Chemistry, Faculty of Pharmacy, Comenius University, Odbojárov 10, 83232 Bratislava, Slovakia

**Keywords:** drug design, targeting, chemical biology, biological chemistry, proteins and nucleic acids, natural compounds, synthesis, biomaterials, nanoparticles, ADME, drug delivery systems

## Abstract

The Eighth Central European Conference “Chemistry towards Biology” was held in Brno, Czech Republic, on August 28–September 1, 2016 to bring together experts in biology, chemistry and design of bioactive compounds; promote the exchange of scientific results, methods and ideas; and encourage cooperation between researchers from all over the world. The topics of the conference covered “Chemistry towards Biology”, meaning that the event welcomed chemists working on biology-related problems, biologists using chemical methods, and students and other researchers of the respective areas that fall within the common scope of chemistry and biology. The authors of this manuscript are plenary speakers and other participants of the symposium and members of their research teams. The following summary highlights the major points/topics of the meeting.

## 1. Introduction

Discoveries and knowledge within chemistry, life and biomedical sciences have been increasingly accelerating; therefore, communication among scientists and research teams around the world is more and more important. One of the possibilities is to get together within various specialised conferences. The Central European Conference series “Chemistry towards Biology” was initiated in 2002 by a meeting in Portorož, Slovenia. The aim of the series is to promote the exchange of scientific results, methods and ideas and encourage cooperation between researchers from all over the world. The topics of the conferences cover “Chemistry towards Biology”, meaning that the events welcome chemists working on biology-related problems, biologists using chemical methods, and students and other researchers of the respective areas that fall within the common scope of chemistry and biology.

The Eighth Central European Conference “Chemistry towards Biology” [1] was held in Brno, Czech Republic, on 28 August–1 September 2016. The eighth year of the conference series was devoted to the following research topics: (i) drug design, research and development; (ii) chemistry of natural compounds; (iii) carbohydrate chemistry; (iv) molecular biology; (v) biochemistry; (vi) biomaterials; (vii) structure, function and interactions of proteins; (viii) engineered enzymes; (ix) nucleic acids chemistry; (x) pharmacology; (xi) drug formulations and drug delivery systems. In total, 136 active participants from 18 countries around the world presented their novel results. The authors of this manuscript are plenary speakers and other participants of the symposium and members of their research teams. The following summary highlights the major points and topics of the symposium. Individual experimental-reviewing contributions/sections are ordered from the biological/chemico-biological point of view to chemical aspects of design and targeting of bioactive compounds. The whole manuscript is closed by hot-topic big data problems in drug design and structure-property studies.

## 2. Disentangling Puzzles: Atomic Resolution Studies of Intrinsically Disordered Proteins

Detailed information about the protein structure is of key importance for the mechanistic understanding of biological activities. As the classical paradigm of structural biology states “one protein–one structure–one function”, it is generally believed that the protein structure and its function are directly interrelated [2]. Although it is commonly true for a large number of proteins, many of them are biologically active without having a unique and stable 3D structure. Those proteins, which in their native conditions sample a multitude of diverse conformational states characterised by high spatiotemporal heterogeneity, are most often termed as intrinsically disordered proteins (IDPs) or natively unfolded (UF) proteins. This class also includes proteins combining well-structured parts with intrinsically disordered protein regions. IDPs are highly abundant in nature and can be found in any given proteome [3]. Conformational states of disordered proteins at the physiological conditions resemble the unfolded state of structured proteins. Currently, it is largely accepted that the intrinsic disorder can have multiple faces and can affect different levels of protein organization. Whole proteins or various protein regions can be disordered to a different degree. No distinct boundary exists between ordered and disordered proteins, as the transition between those two protein classes is rather continuous. With the growing evidence of their important roles in fundamental cellular processes, there is an urgent need to characterise the conformational behaviour of IDPs at the highest possible level. Among all available techniques of modern structural biology, NMR represents the ultimate tool for studies of unstructured or partially disordered proteins at the atomic resolution.

In principle, intrinsically disordered proteins can be studied using a standard set of triple-resonance NMR experiments applied to ^13^C-, ^15^N-labelled samples. However, a combination of the structural disorder with a high incidence of sequential repeats often results in spectra with severely overlapped peaks, impossible to decipher with the traditional approach, as the ensemble averages of the measured chemical shifts are close to their random coil values. Recently, a new NMR methodology emerged, which allows us to significantly shorten experimental time and enhance the spectral resolution needed for a thorough description of unstructured or partially disordered proteins. To facilitate the atomic resolution studies, we have designed a suite of high-dimensional (4D-5D) NMR experiments, which combine ^13^C-direct detection, non-uniform sampling, and non-standard data processing procedures to substantially enhance the attainable resolution and developed protocols for efficient measurements and analysis of the relaxation date, allowing us to characterise motional properties of IDPs.

First, a strategy for complete backbone and side-chain resonance assignment using proton detection was documented on a particularly difficult protein with a highly repetitive sequence, the 20 kDa δ-subunit of RNA polymerase from *Bacillus subtilis* which is unique for Gram-positive bacteria. The protocol is based on three resolution-enhanced NMR experiments: 5D HN(CA)CONH provides sequential connectivity, 5D HabCabCONH is utilised to identify amino acid types, and 5D HC(CC-TOCSY)CONH was used to assign the side-chain resonances. The improved resolution was achieved by a combination of high dimensionality and long evolution times, allowed by non-uniform sampling in the indirect dimensions [4].

Replacement of protons by the carbon (C-13) nuclei of carbonyl groups in the detection scheme offers a possibility to substantially improve the resolution of mD NMR experiments. Applying this concept, we have designed two 5D NMR experiments (CACONCACO, NCOCANCO) for backbone assignment of disordered proteins and successfully demonstrated their performance on the δ-subunit of RNA polymerase. A collection of 0.0003% of the data needed for a conventional experiment with linear sampling within just 24 h was sufficient to perform an unambiguous assignment of the disordered part of the protein from a single 5D spectrum [5].

The developed methodology initiated a thorough structural study of the full-length construct of the δ-subunit of RNA polymerase. The three-dimensional structure of the folded *N*-terminal domain, derived from the observed nuclear Overhauser effects (NOEs), was obtained. The combination of N-15 relaxation data, paramagnetic labelling, and chemical shift analysis resulted in the first picture of the conformational behaviour of the disordered, flexible *C*-terminal domain [6].

In a subsequent study, we developed and tested a pair of 4D NMR experiments, which in addition to the assignment of the backbone resonances also provide correlations of the aliphatic proton (H^α^ and H^β^) and carbon (C^α^ and C^β^) resonance frequencies to the protein backbone. Thus, all the chemical shifts regularly used to map the transient secondary structure motifs in the intrinsically disordered proteins (H^α^, C^α^, C^β^, C′ and N) can be extracted from each spectrum. The experiments were successfully applied to the original assignment of a 12.8 kDa intrinsically disordered protein having a high content of proline residues (26%) in the sequence [7].

To demonstrate that the strategy combining high dimensionality, carbon detection, and non-uniform sampling can be applied to studies of larger IDPs, we have coupled the previously described 5D CACONCACO experiment [5] for the sequential assignment of the backbone resonances, which is not interrupted by the presence of the proline residues in the amino acid sequence, with a novel 5D HC(CC-TOCSY)CACON experiment to facilitate the assignment of the aliphatic side-chain resonances. This approach allowed us to assign backbone atoms of all residues including prolines and 98.2% H-1 and C-13 nuclei in aliphatic side chains of the archetypal microtubule-associated protein MAP2c with the molecular weight of 49.2 kDa [8]. The analysis of the chemical shifts revealed that MAP2c is not completely disordered in the unbound state, but forms transient secondary structure motifs related to its function.

To measure and analyze the relaxation data for characterization of the dynamical and motional properties of intrinsically disorder proteins, novel multi-dimensional NMR experiments were introduced. NMR relaxation provides a valuable insight into molecular motions of both ordered and disordered proteins. However, interpretation of the relaxation data of IDPs has to take into account the lack of a regular structure. Spectral density mapping represents the method of choice, since approaches commonly applied in studies of well-structured proteins cannot be used in case of IDPs. We have developed a new N-15 reduced spectral density mapping protocol relying on the measurements of N-15 relaxation rates in the backbone amide group of 15 N–labelled proteins, which employs cross-correlated relaxation rates. Various sources of possible systematic errors were analyzed theoretically and the presented approaches were tested [9].

Standard spectral density mapping protocols, well suited for the analysis of N-15 relaxation rates, introduce significant systematic errors when applied to C-13 relaxation data, especially if the dynamics are dominated by motions with short correlation times (small molecules, dynamic residues of macromolecules). We have designed a suite of protocols for analyzing C-13 relaxation data and tested their performance. Applicability of the proposed protocols was documented in two case studies, spectral density mapping of a uniformly labelled RNA hairpin and of a selectively labelled disaccharide exhibiting highly anisotropic tumbling. The combination of auto- and cross-correlated relaxation data acquired at three magnetic fields was applied in order to separate effects of fast motion and conformational or chemical exchanges in the case of an RNA hairpin. An approach using auto-correlated relaxation rates acquired at five magnetic fields was used in the case of a selectively labelled disaccharide [10].

To conclude, the recently developed high-dimensional experiments have greatly facilitated the assignment of resonances in IDPs. Despite only small deviations of the observed chemical shifts from their random coil values, the introduced approaches make manual analysis of the spectra very intuitive and straightforward and automated data analysis easy to implement. Most importantly, the above-mentioned experiments achieve a resolution sufficient to provide data for all residues in an IDP sequence. As, in principle, the molecular weight of IDPs suitable for NMR studies is not constrained by their hydrodynamic properties, the practical size limits have yet to be explored. Our recent paper “Toward optimal-resolution NMR of intrinsically disordered proteins” reviewed the overall progress of the field in more detail [11].

## 3. Amyloid Fibril Formation in Details: Dead-End Street of Protein Folding?

Out of the ~100,000 proteins of a eukaryotic cell, ~70% are built up from domains and modules of autonomous 3D-folds. Several known diseases are related to protein domain misfolding proceeded amyloidogenesis, where globular proteins misfold and thus misassemble, making insoluble and toxic oligomeric and polymeric cross-β-sheet fibrils, called amyloids [12]. They have been found to be a result of the formation of amyloid aggregates that are practically independent of the original primary sequence of the protein. Consequently, the driving force of the transformation from the original to disordered amyloid fold is expected to lie in the protein backbone, which is common to all proteins [13]. However, the exact explanation for the existence of such a “dead-end” structure is still unknown. Using systematic first principle calculations on carefully selected but large enough systems modelling the protein backbone, we have shown that the β-pleated sheet structure, the building block of amyloid fibres, is the thermodynamically most stable supramolecular arrangement of all the possible peptide dimers and oligomers both in vacuum and in aqueous environments [13]. Even in a crystalline state (periodical, tight peptide attachment), the β-pleated sheet assembly remains the most stable superstructure. These applied quantum chemistry modellings explain and strongly support why proteins can conclude in the amyloid state regardless of their actual side-chain composition by simply choosing the appropriate conditions. Our quantum chemical modelling (QM) revealed why local structural preferences jeopardise the functional native fold and why the β-pleated sheet-like structure is preferred over any other backbone arrangement.

Studying the structure and internal dynamics of folded (F-), intermediate (I-), unfolded (U-) and amyloid (Amy-) states of polypeptides and proteins is, however, a challenge for several experimental reasons. Even for the smallest folded (mini)proteins, such as the Trp-cage fold Tc5b [14] which comprises just about 20 amino acid residues, acquiring specific diffraction and spectroscopic information is difficult, as often states coexist at very different ratios and exchange at a various timescale of motion. Attempts are made to decipher and better understand key structures, on-pathway intermediates and structural triggers of vital protein transformations such as amyloid formation, F→Amy, as well as those protein unfolding pathways (F→U) that exclude aggregation. Beside conventional tools and spectroscopic methods, the improvement and development of new methodologies, especially those applicable in the field of conformational ensembles, such as CCA+ (a spectral deconvolution method of high plasticity) [15,16,17] or het-NMR (T-dependent ^1^H-^15^N/^13^C-HSQC)-based intermediate I-state characterization [18] for better understanding the driving forces of exchanging F↔U, F↔CC, F↔Amy partners is a must.

**Figure 1:**
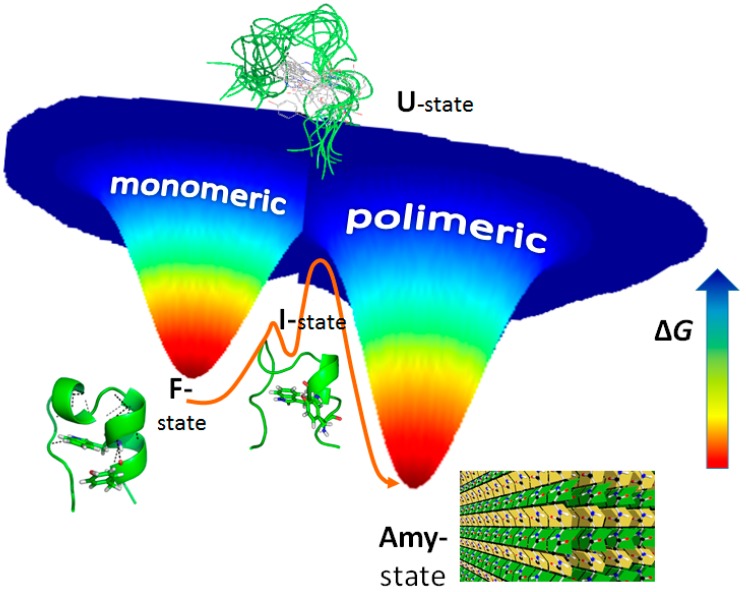
Schematic Δ*G* profile of F↔I↔U protein folding↔unfolding pathway with amyloid state, Amy, available via a key I-state(s).

We have recently acquired key spectroscopic (NMR, CD, FTIR) data, MD results and transmission electron microscopy information on amyloid and fibril morphology on the consequences of serine side-chain phosphorylation of Tc5b [19]. It was shown that the native folded state of Tc5b is destabilised by serine phosphorylation and that the resultant highly dynamic structural ensembles tend to form amyloid-like ordered aggregates of high intermolecular β-structure content. In other words, as predicted by QM studies, if the F-state is destabilised (here by phosphorylation), the population of some I-states emerges and, thus, the Amy-state could become overwhelmingly stable (Figure 1).

By better understanding stability, inter-molecular interactions and the pliability of these nanosystems, the basis of designing and synthesising new products would be granted. Special emphasis on being both able to exclude or, if needed, to utilise biophysical transformations such as amyloid formation would bring in a new era of material sciences and medical research. The significance of understanding the basic principles that preserve protein stability and evoke unfolding of the macromolecules that constitute living systems cannot be overstated. This is especially true today, when biopharmaceuticals are gaining considerable weight in drug research (share of a hundred billion USD/year), so peptides and proteins are increasingly used as medicines. Macromolecules associated with type II diabetes, Alzheimer’s disease, and Creutzfeldt-Jakob syndrome will be at the centre of research studies, but also nano-designs of tightly packed protein segments will be elaborated, resulting in biocompatible nanomaterials such as molecular glues, vehicles, or nano-carrier systems.

## 4. Specific Roles of Histidyl and Cysteinyl Residues in Metal Ion Binding Sites in Peptides and Proteins

The prevalence of various amino acids in proteomes of living organisms varies within a range of 1–10% [20]. Analysis of many proteins has shown that in some cases a given amino acid may occur more frequently, generating a domain rich in one type of amino acid. This typically occurs when a protein or its specific fragment is responsible for the performance of unique functions. Literature data suggests that 2% of all proteins contain regions with at least a six-fold repetition of one amino acid. Usually these are Glu, Ala, Asp, Gly and Ser residues. The repetitions of these amino acids in protein domains are also extremely important for the proper functioning of the human body, e.g., occurrence of numerous glutamyl repetitions in some proteins is connected with neurodegenerative diseases [21,22].

Despite the fact that amino acid residues such as cysteine or histidine are not too frequent in protein sequences in comparison to the mentioned residues, they play a key role in the binding of metal ions necessary for many living organisms (e.g., Zn(II), Ni(II) and other metals). What is very interesting, recent studies have shown that domains with histidine repeats are also found in nature—more than 2000 proteins have histidine-rich regions, with about 10% of them having motifs with more than 5 His consecutive residues [23]. Such sequences are found in chaperones of urease- and hydrogenase-utilising species, in Zn(II) transporters, prion proteins, His-rich glycoproteins, venoms of some African snakes or numerous copper-binding proteins [24]. Domains with His repeats are known as His-tag motifs. The name ‘His-tag’ comes from a synthetic tag commonly used in Immobilized-Metal Affinity Chromatography for purification of recombinant proteins (usually, in this technique, hexa-His-tags are used, connected to the *N*- or *C*-terminus of a purified protein, and free sites of immobilised metal ions, e.g., Ni(II), interact with histidine residues in the His-tag) [25]. Examples of natural proteins with His-tag motifs are: histidine-rich metal-binding polypeptide (Hpn, ^10^GHHHHHHHTHHHHYHGGEHHHHHHSSHH^37^) pHpG from *Atheris squamigera* (EDDHHHHHHHHHGVGGGGGGGGGG) and cyclin T1 from the human genome (HPSNHHHHHNHHSHKHSH). The biological function and mechanism of action of these proteins is not yet fully understood.

Detailed analysis has shown that multiple His residues along a poly-His-tag domain bind metal ions in a very effective way, forming polymorphic states. Metal ions, e.g., Cu(II), can bind to various sets of imidazoles depending on the number of histidine residues that are located in these domains with different efficiencies. In a hexa-His-tag, the metal ion is coordinated by a maximum of two His residues, forming six different binding states. A model, in which copper is bound to the first and fifth imidazole nitrogen, is the most stable complex. MD and DFT calculations show that metal ions induce the formation of a regular α-helix structure in this complex [26]. Similar effects were observed in case of the nona-His-tag from pHpG peptide (pHG), but the number of polymorphic states, the stability of the complexes and the impact on the formation of the secondary structure are much higher than in the hexa-His-tag [27]. There is a pronounced correlation between the number of histidines in a His-tag and the secondary structure formation, the polymorphic states and the thermodynamic stability of these complexes. It is also interesting that peptides with His-tag motifs form extremely stable complexes in comparison with other peptides rich in histidine residues, but separated by other amino acids [26,27,28]. Experimental and computational evidence shows that unique properties of the histidine-tag sequences may be extremely important for the biological behaviour of many peptides and proteins, which contain motifs of this type. These domains can play the role of a so called ‘sponge’, storing metal ions. Moreover, mentioned features can be also extremely important in case of snake venoms, stored in the gland venom of snakes without causing damage (inhibitory action of metalloproteinases responsible for haemorrhagic activity) [29,30].

Proteins encompassing poly-Cys regions play an important role in the function of living organisms. They can be found, for example, in Cysteine-Rich Secretory Proteins (CRISPs). The presence of sulphur in the side-chain groups of Cys may be an origin of redox activity of biomolecules containing these residues. Besides its redox properties, Cys plays an important role as anchoring groups for metal ions [31]. The typical role of poly-Cys proteins is the maintenance of metal ion (Zn(II) or Cu(I)) homeostasis and the detoxification of toxic metal ions (e.g., Cd(II) or Ni(II)) [32]. Different numbers of repeats and patterns of Cys-rich regions can be involved in metal coordination (Zn(II), Cd(II) and Ni(II)) and specific relations between the binding sequence, thermodynamic stability and biological function can be found [33]. Studies on mutants of the poly-Cys sequence of the loop domain of HypA, a protein responsible for the homeostasis of Ni(II) in *Helicobacter pylori*, showed the role of these residues in the structure and the stability of Zn(II), Cd(II) complexes with Cys-rich domains in the proteins [34].

## 5. Structural Bioinformatics: Route from 3D Biomacromolecular Structure to Biology

### 5.1. Introduction

Structural bioinformatics is the part of bioinformatics (see for example [35,36]) which deals with the analysis and prediction of the three-dimensional structure of biological macromolecules. It is closely related to computational chemistry and computational biology. Although the production of new 3D structural data is fascinating, one needs qualitatively new approaches to extract structurally and (consequently) biologically relevant information from such a huge amount of data. Such information can then be used in searches for biologically active compounds, including drugs.

Part of our research focus is oriented in this direction. We have developed a collection of software tools that contribute to 3D data analysis and its subsequent implications. Among these are PatternQuery [37] for the quick definition and extraction of biomacromolecular fragments, SiteBinder [38] for fast and accurate comparison of these fragments, and MotiveValidator [39] and ValidatorDB [40] for the validation of ligands and non-standard residues. In order to step forward towards biology, one needs to characterise the above extracted and validated data subsets. For these purposes, we offer AtomicChargeCalculator [41] for the fast calculation of partial atomic charges on small molecules, biomacromolecules and their complexes. We also offer MOLE [42,43], a software tool for the detection and characterization of channels and pores in biomacromolecules. All these software tools are accessible from the website [44]. The majority of the software is also available through Protein Structure Database in Europe (PDBe), and through PDBsum, a Web-based database of summaries and analyses of all PDB structures. Both platforms are operated by EMBL EBI, the European Bioinformatics Institute in Hinxton, UK.

### 5.2. Fragment Detection Tool PatternQuery

PatternQuery [45] is a molecular language based on the Python programming language. This language describes biomacromolecular structural fragments using the nature and relationship between atoms, residues, and other structural elements. The individual fragment descriptions in the language (so-called queries) define the composition, topology, connectivity, and geometry of a fragment. Therefore, the PatternQuery language enables us to operate at the 1D, 2D and also 3D structure level. PatternQuery contains close to 120 keywords; examples of several PatternQuery keywords are given below:

- Atoms (X) returns all atoms with the element symbol X
- Residues (R1, R2) returns all residues with the two-letter code R1 or R2
- ConnectedAtoms (F, r) returns all atoms within distance r from fragment F
- Authors (F) returns the authors of the structure containing fragment F
- Weight (F) returns the molecular weight of fragment F

These queries can be also combined into more complex ones. The PatternQuery language describes a fragment in such a way that it can be easily translated into a set of rules. In parallel, our methodology for finding fragments represents a biomacromolecular structure as a molecular graph, where atoms are vertexes and bonds are edges. Searching for a fragment is therefore realised as the detection of sets of atoms which meet the criteria defined in the PatternQuery language description. This methodology is implemented in PatternQuery server, an interactive web application for finding and obtaining a fragment from the whole Protein Data Bank. Depending on the complexity of the defined queries and the amount of data set entries, running the queries may take from a few seconds up to approximately one hour (for the whole Protein Data Bank).

### 5.3. Comparison Tool SiteBinder

This software [46] tool is focused on a comparison (superimposition) of molecules with identical (or very similar) 2D structures, as this type of comparison is very helpful in processing biomacromolecular fragments. The SiteBinder superimposition consists of two interdependent stages. First, it is necessary to find the correspondence (atom pairing) between the atoms coming from different structures. In the second step, the sets of paired atoms (3D points) are fitted together as tightly as possible by a geometrical transformation (optimal fitting).

Our methodology provides two superimposition approaches—a combinatorial approach and a subgraph matching approach. The combinatorial approach first generates a set of all chemically meaningful atom pairings. These pairings are generated in such a way that first the atoms in both fragments are divided into subsets according to their properties. Specifically, the subsets can be created according to a residue name, a residue identifier and an element symbol. Afterwards, all pairings between fragments which connect atoms from the same subsets are generated. Then for each pairing the optimal fit is performed using a state-of-the-art quaternion algebra approach. Finally, the pairing that provides the closest fit is selected, and the fit calculated using this pairing is taken as the result. This approach can only superimpose fragments containing the same number of atoms for each element symbol. The subgraph matching approach first detects the largest subgraph of the two fragments. Afterwards, it generates the atom pairings based on the subgraph. For all the pairings, the optimal fit is calculated again using the quaternion algebra approach. The best fit is then taken as the result.

SiteBinder also enables multiple fragments to be superimposed. Specifically, it implements the superimposition approach published by Wang et al. [47], adapts it to biomacromolecular fragments and combines it with our algorithm for the superimposition of two fragments. This multiple superimposition approach works in two steps. First, each fragment is superimposed onto the first one. Afterwards, an average fragment such as the arithmetic average of the x, y and z coordinates of the corresponding atoms is calculated. Next, all the fragments are superimposed onto the average fragment. The new coordinates of all these superimposed fragments are used as an input to the next iteration of the multiple superimposition approach. The iterative superimposition process ends when a further iteration is not able to improve the fit.

### 5.4. Validation Tool MotiveValidator and Database ValidatorDB

MotiveValidator [48] is a tool which enables the validation of an individual ligand or sets of ligands. ValidatorDB [49] is a database of validation results for all ligands from the Protein Data Bank, updated weekly. These software applications implement the validation methodology called the validation of annotation, which is essential for the validation of ligands and biomacromolecular fragments. The goal of this approach is to evaluate whether the ligand or non-standard residue is denoted (annotated) correctly, i.e., if its structure corresponds to the three-letter code it was assigned in the PDB file format. Specifically, the topology and stereochemistry of the validated molecule are compared to those of a correct molecule (reference molecule, model), and any differences found are reported. MotiveValidator and ValidatorDB include a rich set of such analyses, and therefore they cover the main issues observed in the topology (2D structure) and geometry (3D structure) of ligands. The validation analyses in MotiveValidator and ValidatorDB can be classified into three categories, namely Completeness, Chirality and Advanced analyses. The Completeness analyses attempt to find which atoms are missing, whether these atoms are part of rings, or if the structure is degenerated, i.e., the molecule contains very severe errors. These severe errors may refer to residues overlapping in the 3D space, or atoms which are disconnected from the rest of the structure. The Chirality analyses are only performed on complete structures, and aim to evaluate the chirality of each atom in the validated molecule. We distinguish between several types of chirality errors: on carbon atoms (C chirality), on metal atoms (Metal chirality), on atoms with four substituents in one plane (Planar chirality), on atoms connected to at least one substituent by a higher-order bond (High-order chirality), and all other chirality issues (Other chirality). The Advanced analyses are focused on issues which are not actual chemical problems, but which can complicate further processing and exploration of the data, and thus should be noted. The Substitution analysis reports the replacement of an atom with an atom of a different chemical element. The Foreign atom analysis detects atoms which originate from the neighbourhood of the validated molecule (i.e., have a different PDB residue ID than the majority of the validated molecule), and generally marks sites of inter-molecular linkage. The Different naming analysis identifies atoms whose name in the PDB format is different from the standard convention for the validated molecule. The Zero RMSD analysis reports molecules whose structure is identical (root mean square deviation = 0 Å) to the model. The Alternate conformations analysis detects the occurrence of alternate conformations in the validated PDB entry.

### 5.5. Charge Calculation Tool AtomicChargeCalculator

This tool enables the fast calculation of accurate partial atomic charges. The atomic charges are numbers describing the distribution of electron density in a molecule, thus providing clues to its chemical behaviour. The usual approach to their calculation is an application of quantum mechanics (QM), which provides us with many charge calculation schemes such as Mulliken population analysis, natural population analysis, the atoms-in-molecules approach, the Merz-Singh-Kollman method, etc. [41]. Unfortunately, QM charge calculation approaches are very time-consuming. A markedly faster alternative is to employ an empirical charge calculation approach. The most popular empirical approach is the electronegativity equalization method (EEM), which calculates the charges via solving a system of linear equations (EEM matrix), containing information about atom distances and parameters describing the hardness and polarisability of individual atom types. The EEM is able to mimic the QM charge calculation scheme for which it was parameterised. AtomicChargeCalculator (ACC) [50] implements the EEM method, embeds all published EEM parameters and also enables the utilisation of user-provided EEM parameters. ACC can perform charge calculations for large sets of organic molecules (e.g., ten thousand molecules or more) and it is also able to calculate EEM charges on really large biomacromolecular systems (e.g., close to a hundred thousand atoms). ACC offers two new approaches for EEM calculation on large biomacromolecules (EEM Cutoff and EEM Cover), which work by splitting the EEM matrix into multiple smaller matrices. With the EEM Cutoff approach, for each atom in the molecule, ACC generates a fragment made up of all atoms within a cutoff radius R of the original atom. Thus, for a molecule containing N atoms, the EEM Cutoff approach solves N smaller EEM matrices describing a set of N overlapping fragments from the original molecule. This markedly reduces the complexity and time demands of the algorithm. EEM Cover provides another streamlining of the calculations. It also splits the EEM matrix into smaller matrices, but it only generates fragments for a subset of atoms in the molecule. Therefore, the number of EEM matrices that need to be solved is reduced by at least 50% compared to EEM Cutoff while maintaining high accuracy.

### 5.6. Channel Detection and Characterization Tool MOLE

MOLE is a software tool focused on the detection of channels and pores in biomacromolecules. A channel is a pathway connecting a point inside the biomacromolecule (e.g., an active site) to an exterior one. A pore is a tunnel that passes through the biomacromolecule from one point on the surface to another. Channels and pores play significant roles in many biologically relevant systems. The algorithm for finding channels implemented in MOLE involves seven steps: (i) computation of the Delaunay triangulation/Voronoi diagram of the atomic centres; (ii) construction of the molecular surface; (iii) identification of cavities; (iv) identification of possible channel start points; (v) identification of possible channel end points; (vi) localization of channels; and (vii) filtering of the localised channels.

MOLE enables us to also calculate the geometrical and physicochemical properties of channels. A channel can be viewed as a void volume inside the biomacromolecular structure, and it can be described using the arrangement of residues which surround this empty volume. Highly interesting parts of the channel are its local narrowings, which are referred to as local minima. The global minimum of the channel is then referred to as the bottleneck.

MOLE can provide three types of channel properties—geometrical, chemical and physicochemical. The chemical properties of the channel are focused on the residues which surround the channel. The best known chemical property is the so-called lining residues, which describes the residues which are found in the channel walls. These chemical properties also include local minima residues, bottleneck residues and various derived criteria such as the second layer of the channel (residues directly adjacent to the lining residues), etc.

The geometrical properties of the channel describe its geometry characteristics. Basic geometrical properties are the channel length and the radius of the channel at a specific point. Important points for measuring the radius are the bottleneck and other local minima. Also the 3D position of the centreline (a line composed of points in the centre of the channel) and the profile of the channel are widely used geometrical properties.

The most complex properties are the physicochemical properties. Nowadays, channel discovery methodologies only provide values for a few of them, i.e., hydropathy, polarity, mutability and charge [42]. Hydrophobicity and hydrophilicity are two extremes of a spectrum, commonly referred to as hydropathy, and describe the tendency of a molecule to interact with water. Polarity is the property of a molecule given by the separation of electric charge, leading to the molecule having electric poles. The mutability (or relative mutability) quantifies the tendency of an amino acid to be substituted (mutated) in a protein’s structure. Substitution with similar amino acids generally retains the protein’s function, while substitution with amino acids with different properties may affect the protein’s structure or function. Relative mutability is high for easily substitutable amino acids (e.g., small polar residues) and low for amino acids which play a significant role in the protein structure (e.g., amino acids with substrate-binding or catalytic activity). Charge describes the localization of charged residues in the channel.

### 5.7. Integration of Software into PDBsum and PDBe

PDBsum integrates ligand validation results from ValidatorDB and information about channels, precalculated via MOLE. Protein Data Bank Europe includes a Coordinate Server [51], which is based on PatternQuery and enables the user to extract predefined parts of the PDB entry (e.g., backbone, selected residues, etc.).

## 6. Simulating Ligand-Receptor Interactions: Challenge to Understand the Dynamic Process Using Static Techniques

The affinity of a ligand (L) by its biological receptor (R) will depend primarily on their ability to form the L-R complex. The formation of such a complex is a dynamic process in which there are many intermolecular interactions that stabilise and destabilise the formation of this L-R complex. For many years, medicinal chemists have been kept sleepless trying to make a correct evaluation of the L-R complex formation, and this difficulty continues unsolved until now. This is understandable since an accurate description of this process would allow the design and development of new more specific and more effective drugs, which are principal goals of medicinal chemistry.

The simulation of this essentially dynamic process with current molecular modelling techniques faces a tradeoff: (i) perform these simulations using dynamic techniques which evaluate the intermolecular interactions quite poorly; or (ii) use techniques of quantum mechanics which are much more accurate to evaluate these interactions, but can only assess a static process. Actually, the combined and complementary use of these computational techniques seems to be the best way to get better results.

A comparative study was performed based on the results obtained in eight different biological systems of interest in medicinal chemistry: dopaminergic receptors (D1 and D2) [52,53], beta-secretase (BACE-1) [54], dihydrofolate reductase (DHFR) [55], sphingosine kinase 1 (SphK1), acetylcholinesterase (AChE) [56], proto-oncogene serine/threonine kinase (B-RAF) and DNA gyrase-subunit B (GyrB). The results indicated that the main factors affecting the L-R simulations are: (a) size of the active site at the receptor (length and depth); (b) number of interactions involved in the complex formation; (c) flexibility of the active site and accessibility of the ligand; (d) flexibility of the ligand; (e) types of molecular interactions involved and (f) structural variability of the ligand. These results have also demonstrated that for the simulations of complexes, relatively simple from a structural point of view, simple simulations by using techniques such as docking and molecular dynamics (MD) are enough to obtain a significant correlation with the experimental data. In contrast, in the case of L-R complexes with an intermediate degree of complexity, it is necessary to introduce hybrid MM/QM calculations to obtain correlations with the experimental data. In the case of the L-R complexes possessing a high degree of structural complexity such as, for instance, in the case of SphK1, it is necessary to extend the MD simulations, introduce quantum mechanical (QM) calculations and perform analysis QTAIM (quantum theory of atoms in molecules) [57,58]. Static techniques, such as QM calculations and QTAIM studies, are very useful as complementary tools in the simulations of L-R behaviours for different biological systems, particularly when we are interested to know details of the molecular interactions involved in the formation of the L-R complex.

## 7. From Vibrational Spectroscopy to Nanoscopy of Skin Systems with Nanoparticles

Vibrational spectroscopy, consisting of infrared absorption/reflection spectroscopy and Raman spectroscopy, represents a powerful molecular spectroscopic tool for chemical characterization of different materials from elemental ones to complex biological systems. However, especially in the case of biological samples, both i) the high chemical sensitivity including the ability of trace amount detection and ii) spatially resolved information are essential to elucidate the complexity of systems and the dynamics of biological processes.

Firstly, the surface-enhanced vibrational spectroscopic (SEVS) techniques based on either surface-enhanced Raman scattering (SERS) or surface-enhanced infrared absorption (SEIRA) can be used for detection and/or identification of low amounts of organic/biologically important compounds. The plasmonic metal nanoparticles and/or nanostructures are used for spectroscopic signal enhancement. However, the interaction of metallic nanoparticles with the components of biological systems can affect their native properties. Furthermore, the healing (basically antibacterial and anti-inflammatory) effects of silver and/or gold nanoparticles (AgNPs/AuNPs) are already known, e.g., [59,60]. The vibrational spectroscopic studies of skin systems show that AgNPs/AuNPs influence the permeation properties of outer skin layers and affect the penetration of various organic substances, for example B vitamins or peptides. The effects of quantities and types of NPs on skin penetration characteristics are evident when evaluating the data of multivariate chemometric algorithms, e.g., principal component analysis (PCA), partial least square (PLS) regression and soft independent modelling of class analogy (SIMCA).

Secondly, classical vibrational micro-spectroscopy is limited from the point of view of spatial resolution by the diffraction limit. That means that confocal Raman micro-spectroscopy can go down below a 1 µm lateral resolution with visible laser excitation, while the mid-infrared micro-spectroscopy is limited at the level of 10 µm [61]. Hence, the classical vibrational micro-spectroscopy cannot provide detailed information on nanostructures or even individual molecules. The study of nanostructures at molecular or even atomic resolution is accessible using scanning probe microscopic (SPM) techniques.

Nowadays, we can combine the spatial resolution of SPM techniques with the chemical/molecular specificity of vibrational spectroscopy using the advanced techniques of tip-enhanced Raman spectroscopy (TERS), atomic force microscope infrared spectroscopy (AFM-IR) and scanning near-field infrared microscopy (SNIM) [62,63,64]. TERS combines SPM with Raman spectroscopy and enables both outstanding detection sensitivity down to the single-molecule level and high spatial resolution down to sub-nanometers. In the case of both AFM-IR and SNIM, the source of irradiation is a tunable (e.g., quantum cascade) infrared laser, adjusted to a specific wavenumber for an imaging/mapping experiment. The laser beam is focused to a space under the tip and coupled with tip oscillations. Both AFM-IR and SNIM measurements reveal chemical nano-scaled imaging information about the sample based on the “distribution” of absorption at the selected wavenumber by the molecules which vibrate at the corresponding frequency and are located under the tip while only SNIM further detects the radiation phase shifts as supplementary material characteristics. TERS, AFM-IR and SNIM are studied to be applied for model systems of skin constituents, mainly for the samples related to the *stratum corneum* and various topically applied molecular and nano-systems. In the life sciences, TERS and IR nanoscopy are gaining attention as appropriate label-free and high-resolution (molecular dimension) techniques. A direct impact on pharmaceutical questions is obvious.

## 8. From Dequalinium to Mitochondria-Targeted Pharmaceutical Nanocarriers

In an unprecedented move, the editors of *Science* chose in 1999 a published textbook image taken by pioneering electron microscopist K. R. Porter (1912–1997) as the front cover of their March fifth issue (Vol. 283, March 5, 1999) and an editorial inside declared that “Mitochondria make a comeback”. What has brought this long-known cell organelle back into the limelight of the broad scientific community? Starting in the late 1980s, a series of key discoveries has been made which significantly revitalised the scientific interest in this organelle. First, two papers from 1988, one published in *Science* [65] and the other one in *Nature* [66], revealed for the very first time the link between mitochondrial DNA mutations and neuromuscular/neurodegenerative human diseases. Second, by the mid-1990s mitochondria, by then well known as the “powerhouse” of the cell, also became accepted as the “motor of cell death” [67], reflecting their recognised key role in the complex pathway of apoptosis. Adding the already-known involvement of mitochondria in almost all biochemical pathways, mitochondria emerged at the turn of the century as a prime pharmacological target [68]. However, strategies for the targeted delivery of drugs and DNA to mitochondria were underdeveloped at that time [69]. A serendipitous discovery made at the bench in the summer of 1996 led to the design and development of a large variety of mitochondria-targeted pharmaceutical nanocarriers which have opened new avenues towards the delivery of biologically active molecules to and into mitochondria inside living mammalian cells.

Further, 1,1’-decamethylene bis(4-aminoquinaldiniumchloride), a cationic bolaamphiphile referred to as dequalinium (DQA), has been used for over 50 years as an antimicrobial agent in mouthwashes, lozenges, ointments and paints. The exclusive localization of DQA inside mitochondria was experimentally demonstrated in 1987 [70] while mechanistic aspects of its mitochondriotropism were discussed 20 years later [71]. DQA possesses a wide variety of pharmacological activities as summarised in [72]: K^+^ channels, F1-ATPase, calmodulin, proteinase K and mitochondrial DNA all have been reported as potential molecular targets. In the mid-1990s, during the search for a then-putative DNA gyrase-like topoisomerase activity associated with apicoplast DNA in *Plasmodium falciparum* [73], a large number of compounds known to interfere with DNA metabolism [74] were screened and one of them was DQA. By pure chance it was found that under certain experimental conditions, DQA is able to self-assemble into liposome-like vesicles named at the time of that discovery DQAsomes (DeQAlinium-based lipoSOMES) [75]. The strong affinity of DQA for mitochondria combined with its ability to form nano-sized cationic vesicles led to the proposal of using DQAsomes as the very first potential mitochondria-targeted DNA delivery system [76], the proof-of-concept for which was provided 10 years later with the very first report of a successful functional transgene expression in mammalian mitochondria [77]. In parallel to developing them as a mitochondrial transfection vector, DQAsomes have effectively been exploited in vitro and in vivo as a mitochondria-specific nanocarrier for improving the mitochondria-based proapoptotic activity of small molecules, summarised in [78]. Nowadays, DQAsomes are considered as the prototype of all mitochondria-targeted pharmaceutical nanocarriers [79], the further exploration of which will eventually lead to new treatments of mitochondrial diseases.

## 9. ASPH Inhibitors as Second Generation NOTCH Pathway Modulators

It has been over 100 years since the first observation of notched wings in *Drosophila melanogaster* and the origins of understanding the Notch signalling pathway. Since then, the Notch signalling pathway has been identified as a key pathway in cancer [80]. More recently, therapeutic strategies to address the Notch pathway have been developed, including gamma-secretase inhibitors that prevent the proteolytic processing of Notch family receptors [81]. Unfortunately, gamma-secretase inhibitors suffer from severe, dose-limiting gastro-intestinal toxicity, complicating the clinical potential of this class of agent [82]. Furthermore, in some cancers the Notch pathway can serve as a tumour promoter, while in others is serves as a tumour suppressor [83]. Thus, the context-dependent mechanism of Notch pathway modulation has become a major focus of cancer research.

Aspartyl(Asparaginyl)-Beta-Hydroxylase (ASPH) is a 2-oxoglutarate (2OG) utilizing iron-dependent dioxygenase closely related to epigenetic enzymes such as KDM, TET1-3, and FTO [84]. ASPH catalyzes post-translational hydroxylation of critically positioned aspartic acids and asparagines in specific calcium-binding Epidermal Growth Factor (cbEGF) domains, including most proteins involved in the Notch signalling pathway. Biologically, ASPH is involved in trophoblast invasion of the uterine wall and is expressed in the endoderm of developing embryos, although expression in healthy adult tissue is extremely limited [85]. Experimentally confirmed cbEGF substrates of ASPH include LDLR, C1R, JAGGED1, FX, and computationally predicted substrates include NOTCH1-4, JAGGED1&2, DLL1&4, DNER, DLK1&2 among others. ASPH has been demonstrated to activate the Notch signalling pathway both in vitro and in vivo. Hepatocellular carcinoma and pancreatic cancer are known to significantly over-express ASPH on the cell surface, conferring an aggressive, invasive phenotype. Other cancers such as mammary carcinoma also over-express ASPH. ASPH has been demonstrated to aberrantly activate the NOTCH signalling pathway [85]. ASPH inhibitors have been rationally designed and synthesised, and demonstrate predicted activities in vitro [86], including suppression of migration, invasion, and activation of NOTCH pathway–related proteins. In vivo proof-of-principle experiments demonstrate significant suppression of tumour growth at 1 mg/kg [87]. ASPH inhibitors are orally bioavailable, are not genotoxic, have no identified in vitro safety liabilities, and have not demonstrated intestinal toxicity unlike gamma-secretase inhibitors because of the context-specific nature of ASPH activation of the Notch signalling pathway.

## 10. Characterization and Applications of Cysteine-Histidine–Dependent Amidohydrolase Peptidase Targeting Methicillin–Resistant *Staphylococcus aureus*

*Staphylococcus aureus* is a major cause of infection in humans and animals, causing a wide variety of conditions from local inflammations to fatal sepsis. The bacterium is commonly multi-drug resistant and thus many front-line antibiotics have been rendered practically useless for treating human infections. Bacteriophages (phages) are bacterial viruses which are natural predators of bacteria that infect cells and exploit the cell’s DNA replication machinery to produce progeny phage particles, which are ultimately released from the cell after phage-induced lysis.

The genomes of three staphylococcal phages (namely phages K, CS1 and DW2) were sequenced and a variety of peptidoglycan hydrolase enzymes were identified. Peptidoglycan is the bacterial cell wall material, which gives the bacterial cell its rigidity. One class of enzyme is formed by the bacterial tail tip hydrolases, which naturally have the function of facilitating injection of the phage genome through the cell wall into the cell interior (after phage attachment to the surface). The specific peptidoglycan hydrolases identified in these cases were either lysozymes or cysteine-histidine–dependent amidohydrolase peptidases (CHAPs). Both forms were cloned and purified and shown to be active through the use of zymograms (polyacrylamide gels impregnated with autoclaved staphylococcal cells). Enzyme activity in these cases was correlated with clearing of the zymogram in an area corresponding to the predicted migration of the protein after electrophoresis.

The second group of peptidoglycan hydrolase enzymes is made up of bacteriophage endolysins. These are enzymes that facilitate release of progeny bacteriophages at the end of the lytic cycle, through damaging the peptidoglycan and lysing the host cell. In this case, the hydrolases identified were amidases or CHAPs. The endolysin from phage K, like other Gram-positive endolysins, was found to have a modular organization with three domains, the CHAP, an amidase and a cell wall–binding domain. The latter domain facilitates attachment of the enzyme to the bacterial cell wall, while the former two domains catalyze the degradation of the peptidoglycan, mediating rapid bacterial cell death. Both hydrolase genes were cloned, but unlike CHAP, active amidase could not be identified and this is most likely due to not identifying the correct domain boundaries necessary to generate active enzyme. CHAP was purified by ion exchange chromatography with a typical yield of several milligrams of CHAP from one litre of *Escherichia coli* culture. Addition of the enzyme to a turbid bacterial MRSA culture resulted in elimination of turbidity and this could be visualised on microscope slides. In silico elucidation of the three-dimensional structure of the CHAP domain indicated a net positive charge on the molecule. This property is fortuitous as it facilitates attraction to the negatively charged cell wall of staphylococci, a charge resulting from the presence of teichoic acid moieties. The positive charge is not always associated with endolysins targeting staphylococci.

The CHAP enzyme was used in in vivo studies in mouse models where it successfully eliminated MRSA colonization of the nares of the animals without adverse effects. Subsequent bacteriological analysis confirmed complete elimination of all staphylococcal cells from the animal noses. This is significant given that it has been reported that nasal carriage by the patient is the predominant source of the infection in *S. aureus* bacteremia (180 of 219 patients studied) [88]. An apparent low immunogenicity was observed following real-time PCR analysis of gene expression of various proteins associated with the human inflammatory response in human umbilical vein endothelial cells (HUVECs). The proteins analysed included inter-cellular adhesion molecule 1 (ICAM1), vascular cell adhesion molecule 1 (VCAM1), the transcriptional activator cyclo-oxygenase-2 (Cox-2), chemokine (C-X-C motif) ligand 1 (CXCL1), interleukin 32 (IL32) and interleukin 8 (IL8). This apparent low immunogenicity was supported by observation of a relatively low pro-inflammatory response in human blood from three human subjects using enzyme-linked immunosorbent assays, focusing on several cytokines/inflammatory proteins in peripheral blood mononuclear cells, including interferon gamma, interleukin-1b, interleukin-2, interleukin-4, interleukin-6, interleukin-8, interleukin-12-p70 and interleukin-13 [89].

A high-resolution crystal structure of the enzyme was obtained as native crystals diffracted to a maximum resolution of 1.8 Å (methylmercury derivative crystals diffracted up to 1.7 Å) [90]. One interesting point which the structure showed was that there was a calcium ion coordinated close to the active site. It appears that the calcium ion may play a structural role, helping to maintain the structure of the amino terminal domain and thus its catalytic residues in the correct orientation. The calcium ion binding loop also contains residues that may be in contact with the substrate and thus play a role in determining substrate specificity. A zinc ion was also found to be loosely bound to cysteine residue 54, predicted to be part of the catalytic triad. It is hypothesised that this zinc ion may regulate access of the substrate to the catalytic site.

## 11. Natural Product–Based Drug Discovery Revival

Natural products have always been a valuable source of new drugs and they have played an important role in lead discovery [91]. Despite the traditional successful use of natural products as drug leads, recently the pharmaceutical industry shifted its main focus towards synthetic compounds that can be easy produced and resupplied, are more straight-forward for patenting, and are more easily combined in large compound libraries suitable for high-throughput screening (HTS) approaches [92]. However, the combinatorial synthetic chemistry and HTS approaches adopted recently by most pharmaceutical companies did not meet the expectations for improved drug discovery efficiency, and today industrial stakeholders do not see drug discovery strategies with synthetic compounds as superior any more, whereby the potential of natural products as drug leads is again highly appreciated [93]. In line with this notion, the comparative number of scientific studies in the area of natural products pharmacology is rapidly increasing [91,94]. Interdisciplinary approaches, academia-industry partnerships, and virtual screening methods are often stated to be valuable and promising approaches that could help to better harvest the potential of natural product drug discovery [93].

**Figure 2:**
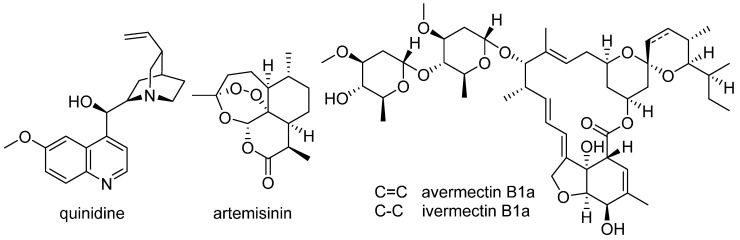
Chemical structures of natural antimalarial compounds and natural and synthetic anthelminthics.

Considered emblematic for the revival of natural product drug discovery might be the 2015 Nobel Prize in Physiology or Medicine, which was awarded to Youyou Tu, William C. Campbell, and Satoshi Ōmura for the discovery of natural products for the treatment of tropical parasitic diseases. Youyou Tu received half of the Nobel Prize “for her discoveries concerning a novel therapy against Malaria” [95]. Malaria is a fatal tropical parasitic disease. In the year 2015, an estimated number of 214 million humans were infected worldwide and 438,000 people died of this disease [96]. Natural remedies to treat malaria have a long history. Cinchona (fever) tree bark has been used traditionally by indigenous South Americans. Quinidine (Figure 2), the bioactive constituent, is an effective antimalarial drug; however, quinidine resistance is a worldwide problem [97]. Youyou Tu discovered the antimalarial sesquiterpene lactone artemisinin (Figure 2) from the traditional Chinese medicinal herb *Artemisia annua* based on ethnomedicinal investigations. Artemisinin brought an important breakthrough in the treatment of malaria and has been used in the clinic since the 1990s. Today, artemisinin-based (combination) therapies of malaria are recommended [95,98,99]. The discovery of artemisinin also opened the path to metabolic engineering of natural products from plants. Although *A. annua* is a reliable source of artemisinin, heterologous generation of artemisinic acid in engineered yeast, followed by transformation into artemisinin by semisynthesis, is performed today to avoid dependency on crop yields and price fluctuation [100]. William C. Campbell and Satoshi Ōmura received the other half of the Nobel Prize “for their discoveries concerning a novel therapy against infections caused by roundworm parasites”, in particular for the discovery of the anthelminthic macrocyclic lactone avermectin, isolated from a soil microorganism, *Streptomyces avermitilis*, and its semisynthetic derivative ivermectin (Figure 2). This discovery has established effective therapies for the treatment of lymphatic filariasis (elephantiasis), which is caused by different parasitic worms and afflicts more than 100 million humans, and onchocerciasis (river blindness), a leading infectious cause of blindness caused by the filarial worm *Onchocerca volvulus*, with which 25 million humans are infected worldwide [95,98,99].

Taken together, although natural product drug discovery often requires more efforts compared to HTS and combinatorial chemistry (e.g., the chemical structures of natural products are often much more complex than usual compounds which chemists synthesise), nature is still considered as the most productive source of promising drug leads for new medicines [93].

## 12. Cardiac Glycosides as Novel Modulators of Cancer Cell Survival

Cardiac glycosides (CGs) are compounds clinically prescribed to treat cardiovascular diseases, with additional anticancer activities. Recent findings corroborate the hypothesis that CGs represent a class of compounds acting on cancer cell signalling and death [101]. UNBS1450, a semi-synthetic cardenolide, exerts cytostatic and cytotoxic effects in leukaemia and solid tumours. In leukaemia, downregulation of proteins with a short turn-over including c-Myc and Mcl-1 was observed. Mcl-1, for instance, appears to act as a universal target for CGs in cancer cells whose proteasome-dependent downregulation eventually leads to apoptotic cell death. UNBS140 also triggers autophagy and mitophagy essentially in solid tumours including neuroblastoma. So far, UNBS1450 was shown to trigger both complete and incomplete mitophagy in neuroblastoma, leading to necroptosis and apoptosis, respectively [102]. Accordingly, CGs can be considered as pharmacological agents allowing cancer cells to switch from one cell death modality to another. The effects appear ubiquitous on different cancer cell models. These findings encourage the further exploration of the potential for CGs in general as cancer cell death modulators alone or in combination with other targeted treatments.

## 13. Antifungal Styrylquinolines as Efflux Pump Inhibitors

*Candida albicans* is one of the most common fungal pathogens in humans [103]. For many years it was the cause of topical and easily curable infections. Nowadays, however, *C. albicans* is commonly present in more dangerous and difficult-to-treat internal organ infections. In immunocompromised patients it is also responsible for life-threatening systemic candidiasis. Additionally, *C. albicans* has the tendency to gain resistance to commonly used antifungals, such as azoles. Drug-resistant strains also became more prevalent recently. One of the main mechanisms of resistance is overexpression and activity of ABC (ATP-binding cassettes) transporters, Cdr1p and Cdr2p, which actively export xenobiotics out of the cell [104,105]. Among them, Cdr1p is the most important in gaining resistance to azole drugs being overexpressed in the majority of drug-resistant strains [106]. With this in mind, the compounds interacting with efflux pumps are especially interesting as candidates for novel drugs. Inhibitors of ABC proteins may be efficient adjuvants for breaking through the drug resistance in persistent infections.

**Figure 3:**
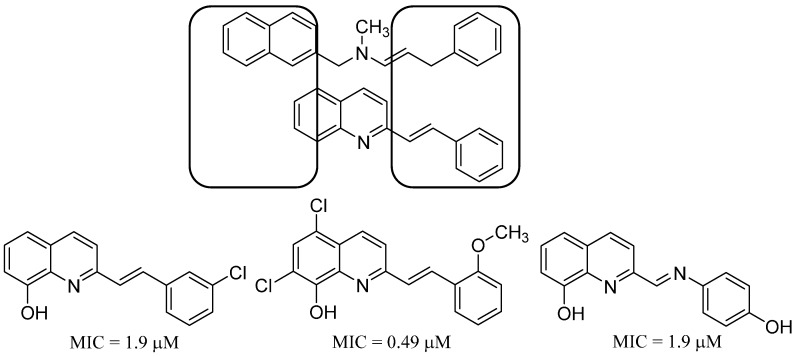
Antifungal styrylquinolines [108].

Quinoline derivatives are well known for their biological activities including antimicrobial and anticancer fields. Due to high prevalence among drug molecules, alkaloids and bioactive substances, quinoline has been claimed to be a privileged structure [107]. Styrylquinolines—lipophilic and rigid analogues of allylamines, see Figure 3—are an interesting example of quinoline-based antifungals [108]. Styrylquinolines have an interestingly wide spectrum of activity, covering antimicrobial and antiproliferative potency [108,109,110,111]. Due to their structural features as a flat and rigid skeleton of aromatic carbon atoms they revealed a tendency to accumulate in cellular membranes and specific organelles. In the present study we investigated the fate of styrylquinolines in the fungal cell to reveal the plausible mechanism of action.

Preliminary screening showed inhibition of the *C. albicans* growth by those compounds and their synergistic activity with fluconazole. The Δ*CDR1* mutant was more sensitive to tested compounds. This result may indicate that styrylquinolines are a substrate for the Cdr1p pump which we confirmed using the rhodamine 6G assay. Additionally, using GFP-tagged Cdr1p, we showed that styrylquinolines induce expression of this transporter. After 4 h of incubation with the compounds, we observed partial delocation of GFP fluorescence from the plasma membrane to the cytoplasm. A similar effect was observed after incubation with amphotericin B and filipin, both compounds that bind to ergosterol and destabilise the cell membrane.

## 14. Selected Chemical and Biological Applications of Pyridine-Appended Click Triazole Derivatives

Despite the fact that 1,2,3-triazole has been known for more than a century [112], the number of its applications has recently increased dramatically. This is mainly due to the discovery that this heterocycle can be easily and selectively prepared by a copper-catalysed cycloaddition between organic azide and terminal acetylene, also known as the click reaction [113,114,115]. Thus, 1,2,3-triazole has emerged as a privileged heterocycle in a wide variety of research areas including material, pharmaceutical and biological sciences [116]. Applications in bio-conjugation [117], sensing events [118], ions binding and transport [119], and polymer chemistry [120] are notable. Its derivatives possess antimicrobial, antibacterial, fungal, anti-inflammatory, analgesics, anti-HIV, anti-allergic, antineoplastic, antianxiety, and anticancer activity, among others [121].

A special interest in coordination and organometallic chemistry has been devoted to the coordinating properties of 1,2,3-triazoles and its derivatives. The 1,2,3-triazole itself can bind to a metal by a variety of different modes, rapidly increasing by the introduction of substituent(s) that can serve as an additional ligand(s) to the metal centre [122]. Thus, 1,2,3-triazole that is functionalised by pyridine became an important chelating ligand [123].

We have been interested in click 1,2,3-triazoles tethered to pyridine as well as pyrimidine, pyrazine and quinolinone rings [124,125]. These molecules have been studied as versatile coordination ability ligands for platinum, palladium, copper, ruthenium, rhodium, silver, gold, mercury, and others, enabling supramolecular associations [126,127]. We have shown that ruthenium complexes of this type have cytotoxic activity against different tumour cell lines in vitro [128]. One of the examined complexes was found to be more cytotoxic than cisplatin against human lung squamose carcinoma cell line (A-549). Pyridine-appended 1,2,3-triazoles have been prepared as diazenecarboxamide-extended cisplatin and carboplatin analogues for a potential combined drug therapy against cancer [129,130,131,132]. One of the products showed cytotoxic activity against human cervical carcinoma HeLa cells that was similar to cisplatin [133].

Alkylation of 1,2,3-triazole leads to the formation of the corresponding triazolium salts, which can serve as precursors for 1,2,3-triazolylidene ligands in organometallic chemistry. A special challenge in alkylation of the 1,2,3-triazole ring is posed when other potentially nucleophilic groups are present in the molecule, as it is in the case of pyridine-appended triazoles. We have recently developed a highly selective protocol for the preparation of 1,4-disubstituted-3-methyl-1,2,3-triazolium salts [134], which found application in organometallic chemistry as precursors for 1,2,3-triazol-5-ylidene ligands, an interesting class of chelating pyridyl-mesoionic carbene ligands. Their complexes with transition metal ions have been investigated as homogeneous catalysts in organic chemistry [135,136,137,138]. Remarkably, a palladium complex of pyridine-appended 1,2,3-triazolylidene catalysed the copper-, amine-, phosphine-, and additive-free aerobic Sonogashira alkynylation of (hetero)aryl bromides in water as the only reaction solvent [139]. This complex has been employed in “green” synthesis of a precursor of SIB-1508Y (altinicline), a potential drug for neurodegenerative diseases [140].

Ruthenium and osmium complexes with 1,2,3-triazolylidene monodentate ligands have been shown to possess a promising cytotoxicity against tumour cells [141]. Interestingly, we have demonstrated that the precursor 1,2,3-triazolium salts are already cytotoxic against several different tumour cell lines as well as carboplatin- and cisplatin-resistant sublines. The cytotoxicity is cell type–dependent and significantly higher against tumour cells than normal cells [142].

## 15. Big Data Problem in Drug Design and Structure-Property Studies

Chemistry attempts to find the rules that control the behaviour of chemical compounds. Preferably for the universal laws, this refers to a whole population of molecules and/or substances, e.g., conservation energy law. On the other hand, classical QSAR (QSPR) is used to describe a small series of congeneric compounds. With the enlargement of the chemical space we could have modified the questions asked. For example, we got interested in if a general rule exists that differentiates drugs from non-drugs, which molecular descriptors decide this or what does drug-likeness mean. At the same time, the availability of computers resulted in the explosion of information. Accordingly, we realised that data became big recently. There are many definitions of big data but generally what decides the difference between the conventional and big datasets are volume, velocity and variety, where volume refers to a massive size of datasets, velocity to the rate of the information increase, and variety to the diverse data forms here [143]. Alternatively, big data is sometimes defined by high information complexity where traditional methods fail when used for processing. How should big data be gathered and managed? What questions to ask in order to address and answer real problems, in particular in drug design? How important are big data here?

It is generally believed that big data brings new value and innovation. For example, Szlezak et al. cited the recent McKinsey research that suggests that the potential use of big data in US health care could reduce costs by $300 billion a year [144]. However, this kind of information is also much less clearly defined and messy. Accordingly, its analysis causes serious problems. The first is that conventional statistics is designed for conventional data. We are not aware enough of the differences and we are not ready to change the addiction to small data sets.

With, datafication, i.e., the tendency of the growing importance of data acquisition and management, we need to better understand the structure of molecular data population in chemistry and drug design. In particular, the chemical space is a structure for molecular data manipulation, where chemical compounds are represented by molecular descriptors (the parameters that can be calculated for the molecules in silico) or properties which should be measured in real non-silico, e.g., in vitro experiments [145,146,147,148]. Let us address the question of the data type that we can encounter as big records in drug design. Let us define the data simply as the collection of information formed by records. This can grow big both by an increase of the number of objects, by the increase of the number of variable entries describing an individual object or by the increase of both the objects and observables. We can further observe that there are several basic big data types, i.e., properties measured for factual chemical space (FCS) substances, properties predicted for FCS or virtual chemical space (VCS) substances or descriptors calculated for FCS or VCS molecules [148]. Despite the common belief, measured properties are rare in the chemical space [143]. Accordingly, the traditional QSARs are based on the expansion of the descriptor data block. It is just recently that properties are being measured for larger populations of chemical compounds, namely in combinatorial chemistry, polypharmacology or omics approaches. PASS [149] is an early example where we realised a fact of a property deficit, therefore, measuring or predicting much larger property populations was needed.

What are the differences between the traditional QSAR and big data statistics in the context of the model’s descriptive, predictive or prescriptive ability? In particular, this can be illustrated by a case study attempting to answer the question: how much does a molecule cost [150]? The relationship between the structure and a property of a chemical compound is an essential concept in chemistry guiding drug design. Actually, however, we need economic considerations to fully understand the fate of drugs on the market. We have recently reported for the first time quantitative structure-economy relationships (QSER) for a large dataset of a commercial building block library of over 2.2 million chemicals that shows that, on average, what we are paying for is a quantity of matter. Synthetic availability scores and selected atom counts also matter here [150].

The structure-price relationship modelled differs from the classical QSAR in the fact that it is valid for the incredibly high numbers of objects; therefore, it is hard to believe that can be as precise and predictive as the classical QSAR relationship involving small data sets. In fact, this method is a statistic probing the whole population of the molecules available. Accordingly, we propose to use the term molecular statistics to draw attention to this fact [150].
